# Crystal structures of two new divalent transition-metal salts of carb­oxy­benzene­sulfonate anions

**DOI:** 10.1107/S2056989022008295

**Published:** 2022-08-31

**Authors:** Reuben T. Bettinger, Philip J. Squattrito, Darpandeep Aulakh, Christopher G. Gianopoulos

**Affiliations:** a Department of Chemistry and Biochemistry, Central Michigan University, Mount Pleasant, Michigan 48859, USA; b College of Natural Sciences and Mathematics, University of Toledo, Toledo, OH 43606, USA; c Department of Chemistry and Biochemistry, University of Toledo, Toledo, OH 43606, USA; Tulane University, USA

**Keywords:** crystal structure, transition-metal salts, carb­oxy­benzene­sulfonate

## Abstract

Hexa­aqua­nickel(II) bis­(3-carb­oxy-4-hy­droxy­benzene­sulfonate) dihydrate, [Ni(H_2_O)_6_][C_6_H_3_(CO_2_H)(OH)SO_3_]_2_·2H_2_O, (I), crystallizes in the triclinic space group *P*  with the nickel(II) aqua complexes on centers of inversion. The carboxyl­ate group is protonated and neither it nor the sulfonate group is involved in direct coordination to the metal ions. The structure consists of alternating layers of inorganic cations and organic anions linked by O—H⋯O hydrogen bonds that also include non-coordinated water mol­ecules of crystallization. The first-row divalent transition-metal salts of this anion are reported as both dihydrates and tetra­hydrates, with two distinct structures for the dihydrates that are both layered but differ in the hydrogen-bonding pattern. Compound (I) represents the second known example of one of these structures. Hexa­aqua­cobalt(II) bis­(3-carb­oxy­benzene­sulfonate) dihydrate, [Co(H_2_O)_6_][C_6_H_4_(CO_2_H)SO_3_]_2_·2H_2_O, (II), also crystallizes in triclinic *P*  with the cobalt(II) aqua complexes on centers of inversion. The structure is also built of alternating layers of complex cations and organic anions without direct coordination to the metal by the protonated carboxyl­ate or unprotonated sulfonate groups. A robust O—H⋯O hydrogen-bonding network involving primarily the coordin­ated and non-coordinated water mol­ecules and sulfonate groups directs the packing. This is the first reported example of a divalent transition-metal salt of the 3-carb­oxy­benzene­sulfonate anion.

## Abstract 2

The structure of hexa­aqua­nickel(II) bis­(3-carb­oxy-4-hy­droxy­benzene­sulfonate) dihydrate consists of alternating layers of inorganic cations and organic anions linked by O—H⋯O hydrogen bonds that also include non-coordinated water mol­ecules of crystallization. The structure of hexa­aqua­cobalt(II) bis­(3-carb­oxy­benzene­sulfonate) dihydrate is also built of alternating layers of complex cations and organic anions without direct coordination to the metal by the protonated carboxyl­ate or unprotonated sulfonate groups. A robust O—H⋯O hydrogen-bonding network involving primarily the coordinated and non-coordinated water mol­ecules and sulfonate groups directs the packing.

## 1. Chemical context

Over the past two decades, organo­sulfonate and organo­carboxyl­ate anions have received significant attention as building blocks for metal-organic framework (MOF) structures (Dey *et al.*, 2014 ▸; Shimizu *et al.*, 2009 ▸; Cai, 2004 ▸). As part of a longstanding inter­est in metal organo­sulfonate and mixed organo­sulfonate/carboxyl­ate salts (Squattrito *et al.*, 2019 ▸), we have continued this effort with studies of other arene­sulfonates with differing substitution patterns and two structures that resulted from this work are reported here.

## 2. Structural commentary

**Figure 1:**
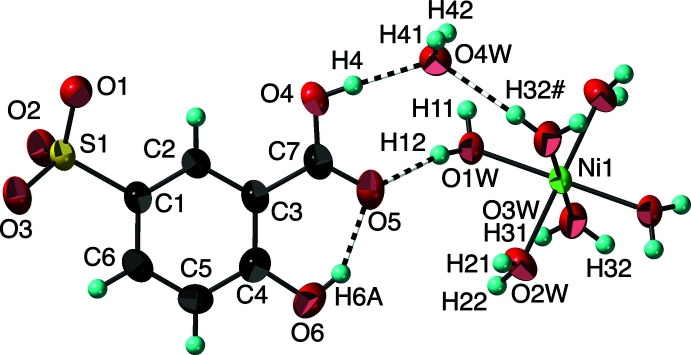
The mol­ecular structure of (I), showing the atom-numbering scheme. Displacement ellipsoids are shown at the 90% probability level and hydrogen atoms are shown as small spheres of arbitrary radii. Hydrogen bonds are shown as striped cylinders. Symmetry-equivalent oxygen atoms are included to show the complete coordination environment of the cation. [Symmetry code: (#) 1 − *x*, 2 − *y*, −*z*]

The product of the reaction of nickel nitrate hexa­hydrate and 5-sulfosalicylic acid (3-carb­oxy-4-hy­droxy­benzene­sulfonic acid) is [Ni(H_2_O)_6_](C_6_H_3_(CO_2_H)(OH)SO_3_)_2_·2H_2_O, (I). The compound crystallizes in the triclinic space group *P* 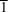 with the asymmetric unit consisting of half a [Ni(H_2_O)_6_]^2+^ cation on the center of inversion, together with one 3-carb­oxy-4-hy­droxy­benzene­sulfonate anion and one non-coordinated water mol­ecule in general positions. As a result of the symmetry, the nickel ion has a very regular octa­hedral coordination of six water mol­ecules (Fig. 1 ▸), with Ni—O distances [2.038 (1), 2.050 (1), 2.053 (1) Å] that are consistent with reported values (Cotton *et al.*, 1993 ▸), including the pattern of one shorter and two slightly longer distances. The O—Ni—O bond angles [87.97 (4)–91.94 (4)°] are within 2° of the ideal. The carboxyl­ate group is protonated and only slightly rotated out of the plane of the phenyl ring [torsion angle C2—C3—C7—O4 = 5.0 (2)°]. The location of the acidic H atom on O4 is unambiguously confirmed on the difference electron-density map and is supported by the C7—O4 [1.318 (2) Å] and C7—O5 [1.236 (2) Å] distances and the hydrogen-bonding pattern (Fig. 1 ▸, Table 1 ▸). The single unique water mol­ecule of crystallization forms four approximately linear strong O—H⋯O hydrogen bonds (Table 1 ▸), the two shown in Fig. 1 ▸ in which the water oxygen atom O4*W* is the acceptor from the carboxyl H4 and a coordinated water mol­ecule [H32#, symmetry code: (#) −*x* + 1, −*y* + 2, −*z*], and two in which the water hydrogen atoms H41 and H42 are donors to sulfonate oxygen atoms O1 and O2, respectively. 
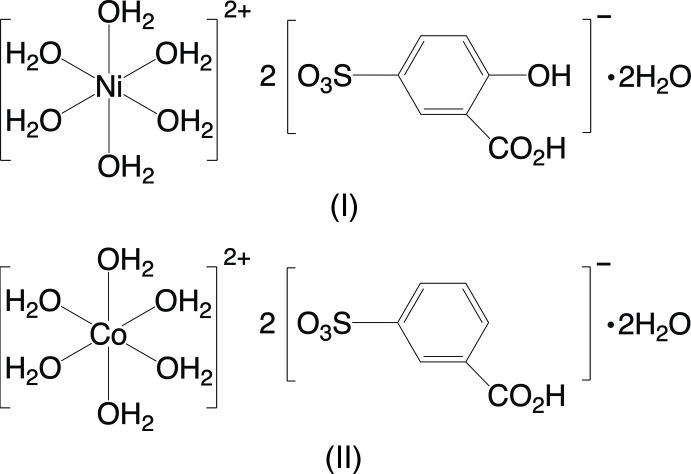


**Figure 2:**
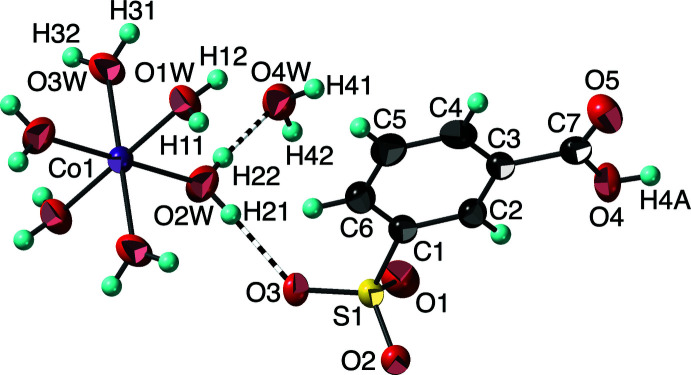
The mol­ecular structure of (II), showing the atom-numbering scheme. Displacement ellipsoids are shown at the 90% probability level and hydrogen atoms are shown as small spheres of arbitrary radii. Hydrogen bonds are shown as striped cylinders. Symmetry-equivalent oxygen atoms are included to show the complete coordination environment of the cation.

The reaction of cobalt nitrate hexa­hydrate and sodium 3-sulfobenzoate (3-carb­oxy­benzene­sulfonate) produced crystals that have been identified as [Co(H_2_O)_6_](C_6_H_4_(CO_2_H)SO_3_)_2_·2H_2_O, (II). Like (I), this compound also crystallizes in the triclinic *P* 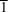 space group with the cobalt cation on the inversion center and the water mol­ecules and 3-carb­oxy­benzene­sulfonate anion in general positions. The hexa­aqua­cobalt(II) ion has a similarly regular octa­hedral coordination with Co—O distances [2.047 (1), 2.092 (1), 2.111 (1) Å] and O—Co—O angles [87.56 (4)–91.15 (4)°] consistent with prior studies (Cotton *et al.*, 1993 ▸). The carboxyl­ate group is unambiguously protonated on O4 [C7—O4 = 1.330 (2) Å *vs* C7—O5 = 1.213 (2) Å] and rotated slightly out of the plane of the ring [torsion angle C2—C3—C7—O4 = 4.6 (2)°]. The sulfon­ate group is rotated about 19° from its position in (I) [torsion angle O1—S1—C1—C2 = −44.42 (13)° in (II) *vs* −25.29 (12)° in (I)]. Presumably this difference is driven by the hydrogen-bonding patterns. The non-coordinated water mol­ecule has a different hydrogen-bonding environment (Table 2 ▸), functioning as an H-atom acceptor from two coordinated water mol­ecules (H22⋯O4*W* is shown in Fig. 2 ▸) and as a donor through H41 and H42 to the carboxyl­ate O5 and the third coordinated water mol­ecule (O1*W*), respectively. These inter­actions are somewhat longer and less linear than those seen in (I).

## 3. Supra­molecular features

**Figure 3:**
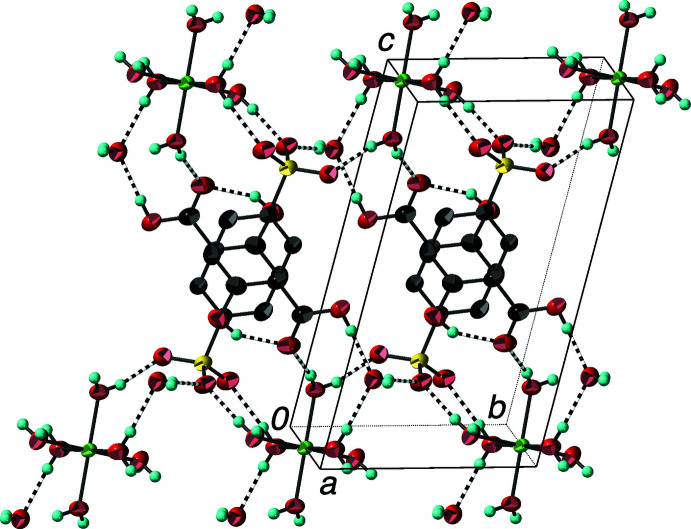
Packing diagram of (I) with the outline of the unit cell. The alternating layers of hexa­aqua­nickel(II) cations and 3-carb­oxy-4-hy­droxy­benzene­sulfonate anions are evident. O—H⋯O hydrogen bonds are shown as striped cylinders. H atoms bonded to C atoms have been omitted. Displacement ellipsoids are drawn at the 90% probability level.

The packing in (I) features layers of hexa­aqua­nickel(II) ions in the *ab* plane alternating with layers of 3-carb­oxy-4-hy­droxy­benzene­sulfonate anions stacking along the *c*-axis direction (Fig. 3 ▸). As has typically been found in related divalent transition-metal arene­sulfonate systems (Leonard *et al.*, 1999 ▸), the anions are inter­leaved in the layer with half having the sulfonate groups directed towards the cation layer above and half towards the cation layer below. The structure also contains a non-coordinated water mol­ecule at the inter­face between the cation and anion layers. The packing is dominated by an extensive network of strong (H⋯O *ca*.1.8–2.0 Å) approximately linear O—H⋯O hydrogen bonds (Table 1 ▸, Fig. 3 ▸) involving the coordinated water mol­ecules, non-coordinated water mol­ecules, and sulfonate and carboxyl­ate groups. All of the water and carboxyl­ate H atoms participate in such an inter­molecular hydrogen bond, while each of the sulfonate and unprotonated carboxyl­ate O atoms function as hydrogen-bond acceptors. The hydroxyl group participates only in an intra­molecular hydrogen bond with the adjacent carboxyl­ate O atom (shown in Fig. 1 ▸).

A hexa­aqua­nickel(II) salt of 3-carb­oxy-4-hy­droxy­benzene­sulfonate has been reported previously (Ma *et al.*, 2003*a* ▸), but unlike (I) it is a tetra­hydrate with two independent non-coordinated water mol­ecules. The extended structure is layered like (I), but differs in the incorporation of the additional water, which results in a modest expansion of the unit cell along the stacking axis *c* and changes to the triclinic cell angles. The [*M*(H_2_O)_6_](C_6_H_3_(CO_2_H)(OH)SO_3_)_2_·4H_2_O structure has also been reported for cobalt (Ma *et al.*, 2003*b* ▸) and zinc (Ma *et al.*, 2003*c* ▸). Dihydrates of the formula [*M*(H_2_O)_6_](C_6_H_3_(CO_2_H)(OH)SO_3_)_2_·2H_2_O have been reported for manganese (Ma *et al.*, 2003*d* ▸), cobalt (Abdelhak *et al.*, 2005 ▸), copper (Ma *et al.*, 2003*e* ▸), and zinc (Lamshöft *et al.*, 2011 ▸). The Mn and Co compounds are isostructural, but the structure is not the same as (I). Specifically, the non-coordinated water mol­ecule is situated differently. In (I) it acts as a hydrogen-bond acceptor from the carboxyl H atom and a coordinated water mol­ecule, while acting as an H-atom donor to two sulfonate O atoms. In the reported Mn and Co dihydrates, the non-coordinated water mol­ecule is a hydrogen-bond acceptor from two coordinated water mol­ecules and an H-atom donor to the unprotonated carboxyl­ate O atom and a coordinated water mol­ecule. The copper dihydrate is superficially similar to the Mn and Co analogs, although the hexa­aqua­copper(II) cation has the expected Jahn–Teller distortion. Perhaps as a result of this, the non-coordinated water mol­ecule has yet a different hydrogen-bonding pattern, accepting from two coordinated water mol­ecules but donating to a sulfonate O atom and a coordinated water mol­ecule. Of the reported dihydrates, only the Zn analog appears to have the same structure as (I) based on the space group and unit-cell dimensions. According to the deposited CIF, only a few of the water H atoms were included in the model and only a cursory description of the extended structure is provided in the paper (Lamshöft *et al.*, 2011 ▸). Thus, (I) represents the first complete structure determination of this dihydrate variant. A recent study of the zinc 3-carb­oxy-4-hy­droxy­benzene­sulfonate system (Song *et al.*, 2019 ▸) demonstrates that it is possible to inter­convert the dihydrate and tetra­hydrate structures by exposure to different relative humidities at moderate temperatures (303 or 313 K). This suggests that the structures are close in energy, as are presumably the dihydrate structures.

**Figure 4:**
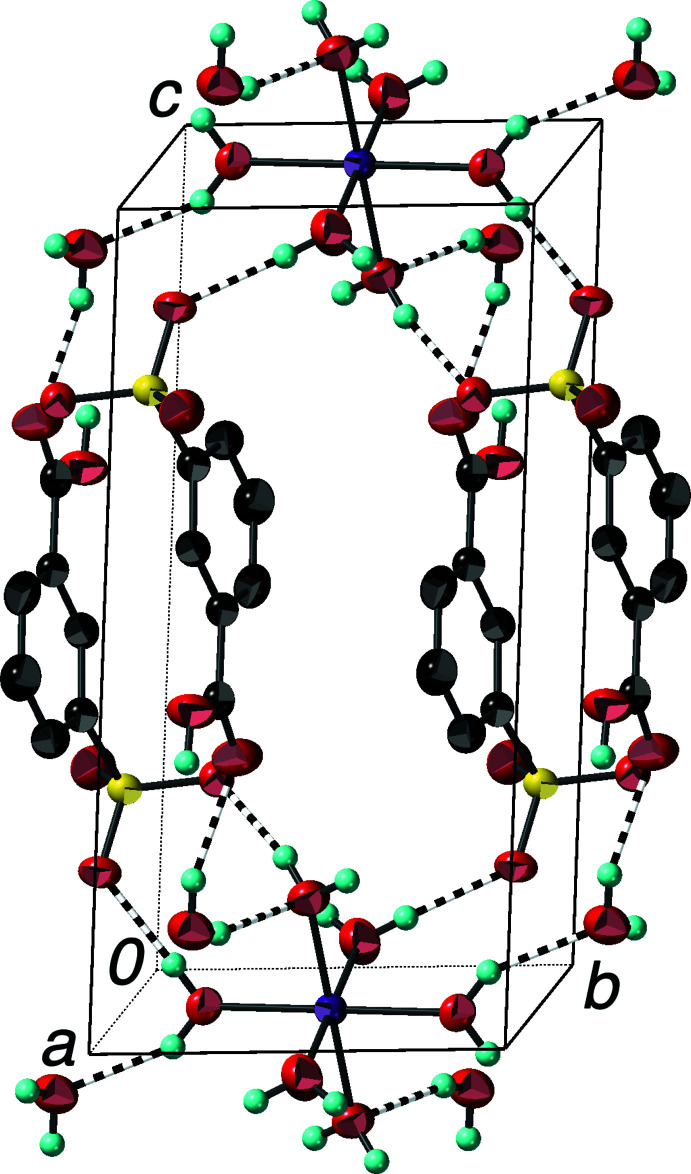
Packing diagram of (II) with the outline of the unit cell showing the alternating layers of hexa­aqua­cobalt(II) cations and 3-carb­oxy­benzene­sulfonate anions. O—H⋯O hydrogen bonds are shown as striped cylinders. H atoms bonded to C atoms have been omitted. Displacement ellipsoids are drawn at the 90% probability level.

The extended structure of (II) is similar to that of (I) with layers of hexa­aqua­cobalt(II) cations in the *ab* plane alternating with layers of inter­leaved 3-carb­oxy­benzene­sulfonate anions (Fig. 4 ▸). Two water mol­ecules per formula unit are found in the inter­face between the layers. The hydrogen-bonding network is somewhat different from that in (I) (Table 2 ▸). The non-coordinated water mol­ecule acts as an H-atom donor to a coordinated water mol­ecule and the unprotonated carboxyl­ate O atom (inter­actions shown in Fig. 4 ▸), and as an H-atom acceptor from the other coordinated water mol­ecules (one of which is shown in Fig. 2 ▸). Other O—H⋯O inter­actions between the coordinated water mol­ecules, the carboxyl­ate H atom, and the sulfonate O atoms complete the hydrogen-bonding scheme. This is the first reported structure of a divalent *d*-block transition-metal salt of 3-carb­oxy­benzene­sulfonate, so it represents a new member of the metal arene­sulfonate family of layered compounds.

## 4. Database survey

A search of the Cambridge Structural Database (CSD, Version 5.42, update of November 2020; Groom *et al.*, 2016 ▸) for the COOH-protonated 3-carb­oxy-4-hy­droxy­benzene­sulfonate ion yielded 21 hits. The ten reported structures containing only metal ions and 3-carb­oxy­benzene­sulfonate ions, with or without water mol­ecules, are tri­aqua­(3-carb­oxy-4-hy­droxy­benzene­sulfonato)­silver monohydrate (refcode FETHES; Gao *et al.*, 2005*a* ▸), penta­aqua-oxo-vanadium(IV) 3-carb­oxy-4-hy­droxy­benzene­sulfonate dihydrate (refcode OBUZUH; Li *et al.*, 2004 ▸), hexa­aqua­manganese(II) 3-carb­oxy-4-hy­droxy­benzene­sulfonate dihydrate (refcode KAGMOV; Ma *et al.*, 2003*d* ▸), hexa­aqua­cobalt(II) 3-carb­oxy-4-hy­droxy­benzene­sulfonate dihydrate (refcode SAYVEU; Abdelhak *et al.*, 2005 ▸), hexa­aqua­cobalt(II) 3-carb­oxy-4-hy­droxy­benzene­sulfonate tetra­hydrate (refcode KAGMUB; Ma *et al.*, 2003*b* ▸), hexa­aqua­nickel(II) 3-carb­oxy-4-hy­droxy­benz­ene­sulfonate tetra­hydrate (refcode KAGNAI; Ma *et al.*, 2003*a* ▸), hexa­aqua­copper(II) 3-carb­oxy-4-hy­droxy­benzene­sulfonate dihydrate (refcode KAGNEM; Ma *et al.*, 2003*e* ▸), hexa­aqua­zinc(II) 3-carb­oxy-4-hy­droxy­benzene­sulfonate tetra­hydrate (refcode KAGNIQ; Ma *et al.*, 2003*c* ▸), hexa­aqua­zinc(II) 3-carb­oxy-4-hy­droxy­benzene­sulfonate dihydrate (refcode FARFOV; Lamshöft *et al.*, 2011 ▸), and bis­(3-carb­oxy-4-hy­droxy­benzene­sulfonato)­diaqua­zinc(II) hydrate (refcode VOJYEB; Song *et al.*, 2019 ▸). Of these structures, only FETHES and VOJYEB feature direct bonding between the sulfonate O atoms and the metal ions, while all of the others are similar to the structures reported herein.

A search of the Cambridge Structural Database (CSD, Version 5.42, update of November 2020; Groom *et al.*, 2016 ▸) for the COOH-protonated 3-carb­oxy­benzene­sulfonate ion yielded 15 hits. The five reported structures containing only metal ions and 3-carb­oxy­benzene­sulfonate ions, with or without water mol­ecules, are silver 3-carb­oxy­benzene­sulfonate at 293 K (refcode ROJJUW; Prochniak *et al.*, 2008 ▸) and 100 K (refcode ROJJUW01; Bettinger *et al.*, 2020 ▸), sodium 3-carb­oxy­benzene­sulfonate dihydrate (refcode ROJJOQ; Prochniak *et al.*, 2008 ▸), bis­muth(III) 3-carb­oxy­benzene­sulfonate tetra­hydrate (refcode LEXKAD; Senevirathna *et al.*, 2018 ▸), and barium 3-carb­oxy­benzene­sulfonate trihydrate (refcode FOBXUQ; Gao *et al.*, 2005*b* ▸). All of these structures feature direct bonding between the sulfonate O atoms and the metal ions with resulting frameworks of varying dimensionalities.

## 5. Synthesis and crystallization

A 2.54 g (10.0 mmol) sample of 5-sulfosalicylic acid (3-carb­oxy-4-hy­droxy­benzene­sulfonic acid) (EMD Chemicals, >99%) was dissolved in 100 ml of water. To this colorless solution was added a green solution of 2.91 g (10.0 mmol) of Ni(NO_3_)_2_^.^6H_2_O (Aldrich) in 50 ml of water. The resulting clear green solution was stirred for about 30 minutes and transferred to a porcelain evaporating dish that was set out to evaporate in a fume hood. After several days, the water had completely evaporated leaving behind large elongated (>1 cm) green slab-shaped crystals, 2.57 g of which were collected by hand from the dish. These were identified as (I) through the single-crystal X-ray study. A 2.24 g (10.0 mmol) sample of sodium 3-sulfobenzoate (Aldrich, 97%) was dissolved in 45 ml of water. To this colorless solution was added a red solution of 2.91 g (10.0 mmol) of Co(NO_3_)_2_^.^6H_2_O (Aldrich) in 50 ml of water. The resulting red solution was stirred for 30 minutes, transferred to a porcelain dish, and set out to evaporate. The final red product was primarily polycrystalline but some small red–pink plates were found to be suitable for single-crystal X-ray analysis, leading to their identification as (II).

## 6. Refinement

Crystal data, data collection and structure refinement details are summarized in Table 3 ▸. Hydrogen atoms bonded to carbon atoms were located in difference electron-density maps, constrained on idealized positions, and included in the refinement as riding atoms with C—H = 0.95 Å and their *U*_iso_ constrained to be 1.2 times the *U*_eq_ of the bonding atom. Oxygen-bound hydrogen atoms were located in difference electron-density maps and refined with isotropic displacement parameters while the O—H distances were restrained to 0.84 (1) Å.

## Supplementary Material

Crystal structure: contains datablock(s) I, II, global. DOI: 10.1107/S2056989022008295/mw2190sup1.cif

Structure factors: contains datablock(s) I. DOI: 10.1107/S2056989022008295/mw2190Isup2.hkl

Structure factors: contains datablock(s) II. DOI: 10.1107/S2056989022008295/mw2190IIsup3.hkl

CCDC references: 2202460, 2202459

Additional supporting information:  crystallographic information; 3D view; checkCIF report

## Figures and Tables

**Table 1:** Hydrogen-bond geometry (Å, °) for (I)

*D*—H⋯*A*	*D*—H	H⋯*A*	*D*⋯*A*	*D*—H⋯*A*
O1*W*—H11⋯O1^i^	0.83 (1)	1.98 (1)	2.7895 (14)	165 (2)
O1*W*—H12⋯O5	0.83 (1)	1.92 (1)	2.7432 (14)	168 (2)
O2*W*—H22⋯O2^ii^	0.84 (1)	1.94 (1)	2.7802 (14)	176 (2)
O2*W*—H21⋯O3^iii^	0.83 (1)	2.02 (1)	2.8476 (14)	174 (2)
O3*W*—H32⋯O4*W* ^iv^	0.83 (1)	1.99 (1)	2.8208 (14)	172 (2)
O3*W*—H31⋯O3^ii^	0.83 (1)	2.05 (1)	2.8790 (14)	175 (2)
O6—H6*A*⋯O5	0.84 (1)	1.84 (2)	2.5904 (16)	148 (2)
O4—H4⋯O4*W*	0.84 (1)	1.83 (1)	2.6656 (14)	171 (2)
O4*W*—H42⋯O2^i^	0.83 (1)	1.91 (1)	2.7420 (14)	178 (2)
O4*W*—H41⋯O1^v^	0.83 (1)	1.99 (1)	2.8029 (14)	165 (2)

Symmetry codes: (i) 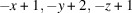 ; (ii) 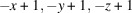 ; (iii) 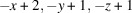 ; (iv) 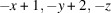 ; (v) 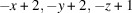 .

**Table 2:** Hydrogen-bond geometry (Å, °) for (II)

*D*—H⋯*A*	*D*—H	H⋯*A*	*D*⋯*A*	*D*—H⋯*A*
O1*W*—H12⋯O2^i^	0.84 (1)	1.94 (1)	2.7757 (15)	170 (2)
O1*W*—H11⋯O1^ii^	0.84 (1)	1.91 (1)	2.7382 (15)	175 (2)
O3*W*—H32⋯O4*W* ^iii^	0.84 (1)	2.04 (2)	2.7887 (16)	150 (2)
O3*W*—H31⋯O3^i^	0.84 (1)	1.95 (1)	2.7852 (16)	178 (2)
O2*W*—H22⋯O4*W*	0.83 (1)	1.92 (1)	2.7516 (16)	174 (2)
O2*W*—H21⋯O3	0.83 (1)	1.96 (1)	2.7925 (15)	176 (2)
O4—H4*A*⋯O2^iv^	0.84 (1)	1.86 (1)	2.6703 (15)	162 (2)
O4*W*—H42⋯O1*W* ^v^	0.83 (1)	2.12 (1)	2.8960 (16)	158 (2)
O4*W*—H41⋯O5^vi^	0.83 (1)	2.08 (1)	2.8503 (16)	153 (2)

Symmetry codes: (i) 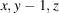 ; (ii) 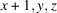 ; (iii) 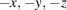 ; (iv) 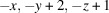 ; (v) 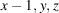 ; (vi) 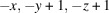 .

**Table 3:** Experimental details

	(I)	(II)
Crystal data		
Chemical formula	[Ni(H_2_O)_6_](C_7_H_5_O_6_S)_2_·2H_2_O	[Co(H_2_O)_6_](C_7_H_5_O_5_S)_2_·2H_2_O
*M*_r_	637.18	605.40
Crystal system, space group	Triclinic, *P* 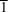	Triclinic, *P* 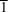
Temperature (K)	150	150
*a*, *b*, *c* (Å)	6.5986 (7), 7.4183 (8), 13.2847 (14)	6.7774 (11), 6.9866 (11), 13.721 (2)
α, β, γ (°)	74.1712 (14), 88.6035 (14), 77.9200 (13)	91.107 (2), 90.401 (2), 117.5832 (19)
*V* (Å^3^)	611.41 (11)	575.66 (16)
*Z*	1	1
Radiation type	Mo *K*α	Mo *K*α
μ (mm^−1^)	1.06	1.01
Crystal size (mm)	0.22 × 0.16 × 0.10	0.30 × 0.14 × 0.08

Data collection		
Diffractometer	Bruker Duo with APEXII CCD	Bruker Duo with APEXII CCD
Absorption correction	Multi-scan (*SADABS*; Krause *et al.*, 2015 ▸)	Multi-scan (*SADABS*; Krause *et al.*, 2015 ▸)
*T*_min_, *T*_max_	0.670, 0.746	0.688, 0.746
No. of measured, independent and observed [*I* > 2σ(*I*)] reflections	8656, 3047, 2779	8197, 2881, 2578
*R*_int_	0.019	0.020
(sin θ/λ)_max_ (Å^−1^)	0.669	0.669

Refinement		
*R*[*F* ^2^ > 2σ(*F* ^2^)], *wR*(*F* ^2^), *S*	0.023, 0.059, 1.08	0.024, 0.062, 1.05
No. of reflections	3047	2881
No. of parameters	209	196
No. of restraints	10	9
H-atom treatment	H atoms treated by a mixture of independent and constrained refinement	H atoms treated by a mixture of independent and constrained refinement
Δρ_max_, Δρ_min_ (e Å^−3^)	0.33, −0.41	0.41, −0.43

Computer programs: *APEX3* and *SAINT* (Bruker, 2015 ▸), *SHELXT2014/5* (Sheldrick, 2015*a* ▸), *SHELXL2018/3* (Sheldrick, 2015*b* ▸), and *CrystalMaker* (Palmer, 2014 ▸).

## supplementary crystallographic information

### (I) Crystal data

**Table:**

[Ni(H_2_O)_6_](C_7_H_5_O_6_S)_2_·2H_2_O	*Z* = 1
*M**_r_* = 637.18	*F*(000) = 330
Triclinic, *P*1	*D*_x_ = 1.731 Mg m^−^^3^
*a* = 6.5986 (7) Å	Mo *K*α radiation, λ = 0.71073 Å
*b* = 7.4183 (8) Å	Cell parameters from 4943 reflections
*c* = 13.2847 (14) Å	θ = 2.9–28.3°
α = 74.1712 (14)°	µ = 1.06 mm^−^^1^
β = 88.6035 (14)°	*T* = 150 K
γ = 77.9200 (13)°	Block, green
*V* = 611.41 (11) Å^3^	0.22 × 0.16 × 0.10 mm

### (I) Data collection

**Table:**

Bruker Duo with APEXII CCD diffractometer	2779 reflections with *I* > 2σ(*I*)
Radiation source: fine-focus sealed tube	*R*_int_ = 0.019
ω scans	θ_max_ = 28.4°, θ_min_ = 2.9°
Absorption correction: multi-scan (SADABS; Krause et al., 2015)	*h* = −8→8
*T*_min_ = 0.670, *T*_max_ = 0.746	*k* = −9→9
8656 measured reflections	*l* = −17→17
3047 independent reflections	

### (I) Refinement

**Table:**

Refinement on *F*^2^	Primary atom site location: dual
Least-squares matrix: full	Secondary atom site location: difference Fourier map
*R*[*F*^2^ > 2σ(*F*^2^)] = 0.023	Hydrogen site location: difference Fourier map
*wR*(*F*^2^) = 0.059	H atoms treated by a mixture of independent and constrained refinement
*S* = 1.08	*w* = 1/[σ^2^(*F*_o_^2^) + (0.0284*P*)^2^ + 0.2223*P*] where *P* = (*F*_o_^2^ + 2*F*_c_^2^)/3
3047 reflections	(Δ/σ)_max_ < 0.001
209 parameters	Δρ_max_ = 0.33 e Å^−^^3^
10 restraints	Δρ_min_ = −0.41 e Å^−^^3^

### (I) Special details

**Table:**

Geometry. All esds (except the esd in the dihedral angle between two l.s. planes) are estimated using the full covariance matrix. The cell esds are taken into account individually in the estimation of esds in distances, angles and torsion angles; correlations between esds in cell parameters are only used when they are defined by crystal symmetry. An approximate (isotropic) treatment of cell esds is used for estimating esds involving l.s. planes.

### (I) Fractional atomic coordinates and isotropic or equivalent isotropic displacement parameters (Å^2^)

**Table:**

	*x*	*y*	*z*	*U*_iso_*/*U*_eq_	
Ni1	0.500000	1.000000	0.000000	0.01105 (7)	
O1W	0.40237 (15)	0.99422 (14)	0.14733 (7)	0.0161 (2)	
H11	0.342 (3)	1.095 (2)	0.1600 (16)	0.039 (6)*	
H12	0.488 (3)	0.939 (3)	0.1966 (12)	0.041 (6)*	
O2W	0.66270 (15)	0.72230 (14)	0.05040 (8)	0.0171 (2)	
H22	0.596 (3)	0.642 (3)	0.0846 (16)	0.048 (6)*	
H21	0.7789 (19)	0.701 (3)	0.0795 (15)	0.037 (6)*	
O3W	0.23500 (15)	0.91381 (15)	−0.02663 (8)	0.0168 (2)	
H32	0.220 (3)	0.884 (3)	−0.0816 (11)	0.037 (6)*	
H31	0.182 (3)	0.840 (2)	0.0200 (12)	0.033 (5)*	
S1	0.78200 (5)	0.52327 (5)	0.79824 (2)	0.01278 (8)	
O1	0.84194 (15)	0.70694 (14)	0.78658 (8)	0.0177 (2)	
O2	0.56747 (14)	0.52970 (14)	0.83170 (8)	0.0176 (2)	
O3	0.92577 (15)	0.35961 (14)	0.86440 (8)	0.0181 (2)	
O6	0.73062 (17)	0.39614 (16)	0.38141 (8)	0.0211 (2)	
H6A	0.704 (4)	0.503 (2)	0.3355 (14)	0.051 (7)*	
O5	0.68377 (16)	0.76283 (15)	0.30157 (8)	0.0204 (2)	
O4	0.72336 (17)	0.94807 (15)	0.40456 (8)	0.0213 (2)	
H4	0.727 (3)	1.025 (3)	0.3454 (10)	0.042 (6)*	
C1	0.78448 (19)	0.48905 (19)	0.67154 (10)	0.0135 (2)	
C6	0.8006 (2)	0.30381 (19)	0.66125 (11)	0.0159 (3)	
H6	0.823672	0.197124	0.721651	0.019*	
C5	0.7830 (2)	0.2757 (2)	0.56350 (11)	0.0171 (3)	
H5	0.793317	0.149826	0.556807	0.021*	
C4	0.7498 (2)	0.4327 (2)	0.47420 (11)	0.0159 (3)	
C3	0.7387 (2)	0.61877 (19)	0.48438 (10)	0.0141 (3)	
C7	0.7129 (2)	0.7820 (2)	0.38932 (11)	0.0160 (3)	
C2	0.7559 (2)	0.64496 (19)	0.58405 (10)	0.0140 (2)	
H2	0.747996	0.769976	0.591539	0.017*	
O4W	0.77781 (16)	1.17956 (14)	0.21815 (8)	0.0167 (2)	
H42	0.674 (2)	1.269 (2)	0.2040 (16)	0.035 (6)*	
H41	0.879 (2)	1.230 (3)	0.2199 (17)	0.039 (6)*	

### (I) Atomic displacement parameters (Å^2^)

**Table:**

	*U*^11^	*U*^22^	*U*^33^	*U*^12^	*U*^13^	*U*^23^
Ni1	0.01121 (12)	0.01190 (12)	0.00927 (12)	−0.00301 (8)	−0.00053 (8)	−0.00113 (8)
O1W	0.0184 (5)	0.0172 (5)	0.0110 (4)	−0.0021 (4)	−0.0004 (4)	−0.0025 (4)
O2W	0.0140 (5)	0.0145 (5)	0.0199 (5)	−0.0035 (4)	−0.0020 (4)	0.0005 (4)
O3W	0.0168 (5)	0.0226 (5)	0.0129 (5)	−0.0095 (4)	0.0003 (4)	−0.0043 (4)
S1	0.01103 (15)	0.01454 (16)	0.01148 (15)	−0.00312 (11)	0.00037 (11)	−0.00112 (12)
O1	0.0193 (5)	0.0183 (5)	0.0175 (5)	−0.0079 (4)	0.0035 (4)	−0.0054 (4)
O2	0.0122 (4)	0.0205 (5)	0.0172 (5)	−0.0030 (4)	0.0032 (4)	−0.0009 (4)
O3	0.0150 (5)	0.0208 (5)	0.0147 (5)	−0.0010 (4)	−0.0024 (4)	0.0001 (4)
O6	0.0239 (5)	0.0249 (6)	0.0174 (5)	−0.0068 (4)	−0.0005 (4)	−0.0093 (4)
O5	0.0211 (5)	0.0251 (5)	0.0132 (5)	−0.0040 (4)	−0.0028 (4)	−0.0024 (4)
O4	0.0306 (6)	0.0159 (5)	0.0142 (5)	−0.0040 (4)	−0.0003 (4)	0.0007 (4)
C1	0.0103 (6)	0.0161 (6)	0.0132 (6)	−0.0030 (5)	−0.0002 (4)	−0.0021 (5)
C6	0.0130 (6)	0.0153 (6)	0.0174 (6)	−0.0038 (5)	0.0008 (5)	−0.0005 (5)
C5	0.0157 (6)	0.0154 (6)	0.0215 (7)	−0.0050 (5)	0.0015 (5)	−0.0058 (5)
C4	0.0101 (6)	0.0219 (7)	0.0166 (6)	−0.0042 (5)	0.0004 (5)	−0.0062 (5)
C3	0.0104 (6)	0.0169 (6)	0.0134 (6)	−0.0027 (5)	0.0002 (5)	−0.0015 (5)
C7	0.0112 (6)	0.0192 (7)	0.0149 (6)	−0.0012 (5)	0.0001 (5)	−0.0014 (5)
C2	0.0124 (6)	0.0141 (6)	0.0145 (6)	−0.0029 (5)	0.0004 (5)	−0.0024 (5)
O4W	0.0160 (5)	0.0164 (5)	0.0178 (5)	−0.0044 (4)	−0.0009 (4)	−0.0039 (4)

### (I) Geometric parameters (Å, º)

**Table:**

Ni1—O1W^i^	2.0376 (10)	O6—H6A	0.843 (10)
Ni1—O1W	2.0376 (10)	O5—C7	1.2364 (17)
Ni1—O2W^i^	2.0498 (10)	O4—C7	1.3179 (18)
Ni1—O2W	2.0498 (10)	O4—H4	0.839 (9)
Ni1—O3W	2.0526 (10)	C1—C2	1.3821 (18)
Ni1—O3W^i^	2.0526 (10)	C1—C6	1.3996 (19)
O1W—H11	0.829 (9)	C6—C5	1.3816 (19)
O1W—H12	0.832 (10)	C6—H6	0.9500
O2W—H22	0.838 (10)	C5—C4	1.4016 (19)
O2W—H21	0.832 (9)	C5—H5	0.9500
O3W—H32	0.833 (9)	C4—C3	1.4098 (19)
O3W—H31	0.831 (9)	C3—C2	1.4003 (18)
S1—O3	1.4570 (10)	C3—C7	1.4762 (19)
S1—O1	1.4644 (10)	C2—H2	0.9500
S1—O2	1.4685 (10)	O4W—H42	0.832 (9)
S1—C1	1.7685 (14)	O4W—H41	0.834 (10)
O6—C4	1.3466 (17)		
			
O1W^i^—Ni1—O1W	180.0	O3—S1—C1	106.97 (6)
O1W^i^—Ni1—O2W^i^	91.26 (4)	O1—S1—C1	106.29 (6)
O1W—Ni1—O2W^i^	88.74 (4)	O2—S1—C1	105.69 (6)
O1W^i^—Ni1—O2W	88.74 (4)	C4—O6—H6A	107.2 (16)
O1W—Ni1—O2W	91.26 (4)	C7—O4—H4	107.2 (15)
O2W^i^—Ni1—O2W	180.0	C2—C1—C6	120.45 (12)
O1W^i^—Ni1—O3W	92.03 (4)	C2—C1—S1	120.22 (10)
O1W—Ni1—O3W	87.97 (4)	C6—C1—S1	119.17 (10)
O2W^i^—Ni1—O3W	88.06 (4)	C5—C6—C1	120.19 (12)
O2W—Ni1—O3W	91.94 (4)	C5—C6—H6	119.9
O1W^i^—Ni1—O3W^i^	87.97 (4)	C1—C6—H6	119.9
O1W—Ni1—O3W^i^	92.03 (4)	C6—C5—C4	120.03 (13)
O2W^i^—Ni1—O3W^i^	91.94 (4)	C6—C5—H5	120.0
O2W—Ni1—O3W^i^	88.06 (4)	C4—C5—H5	120.0
O3W—Ni1—O3W^i^	180.0	O6—C4—C5	117.16 (13)
Ni1—O1W—H11	119.4 (15)	O6—C4—C3	123.10 (12)
Ni1—O1W—H12	117.2 (15)	C5—C4—C3	119.74 (12)
H11—O1W—H12	106 (2)	C2—C3—C4	119.51 (12)
Ni1—O2W—H22	116.1 (16)	C2—C3—C7	121.27 (12)
Ni1—O2W—H21	118.9 (15)	C4—C3—C7	119.21 (12)
H22—O2W—H21	109 (2)	O5—C7—O4	122.61 (13)
Ni1—O3W—H32	119.8 (15)	O5—C7—C3	121.89 (13)
Ni1—O3W—H31	122.3 (14)	O4—C7—C3	115.49 (12)
H32—O3W—H31	106 (2)	C1—C2—C3	120.05 (12)
O3—S1—O1	113.84 (6)	C1—C2—H2	120.0
O3—S1—O2	111.87 (6)	C3—C2—H2	120.0
O1—S1—O2	111.57 (6)	H42—O4W—H41	106 (2)
			
O3—S1—C1—C2	−147.25 (11)	C5—C4—C3—C2	−1.56 (19)
O1—S1—C1—C2	−25.29 (12)	O6—C4—C3—C7	−2.41 (19)
O2—S1—C1—C2	93.39 (11)	C5—C4—C3—C7	177.36 (12)
O3—S1—C1—C6	37.34 (12)	C2—C3—C7—O5	−175.12 (12)
O1—S1—C1—C6	159.30 (10)	C4—C3—C7—O5	5.97 (19)
O2—S1—C1—C6	−82.02 (11)	C2—C3—C7—O4	5.01 (18)
C2—C1—C6—C5	−1.6 (2)	C4—C3—C7—O4	−173.89 (12)
S1—C1—C6—C5	173.82 (10)	C6—C1—C2—C3	1.29 (19)
C1—C6—C5—C4	0.3 (2)	S1—C1—C2—C3	−174.06 (10)
C6—C5—C4—O6	−178.94 (12)	C4—C3—C2—C1	0.28 (19)
C6—C5—C4—C3	1.3 (2)	C7—C3—C2—C1	−178.62 (12)
O6—C4—C3—C2	178.67 (12)		

Symmetry code: (i) −*x*+1, −*y*+2, −*z*.

### (I) Hydrogen-bond geometry (Å, º)

**Table:**

*D*—H···*A*	*D*—H	H···*A*	*D*···*A*	*D*—H···*A*
O1*W*—H11···O1^ii^	0.83 (1)	1.98 (1)	2.7895 (14)	165 (2)
O1*W*—H12···O5	0.83 (1)	1.92 (1)	2.7432 (14)	168 (2)
O2*W*—H22···O2^iii^	0.84 (1)	1.94 (1)	2.7802 (14)	176 (2)
O2*W*—H21···O3^iv^	0.83 (1)	2.02 (1)	2.8476 (14)	174 (2)
O3*W*—H32···O4*W*^i^	0.83 (1)	1.99 (1)	2.8208 (14)	172 (2)
O3*W*—H31···O3^iii^	0.83 (1)	2.05 (1)	2.8790 (14)	175 (2)
O6—H6*A*···O5	0.84 (1)	1.84 (2)	2.5904 (16)	148 (2)
O4—H4···O4*W*	0.84 (1)	1.83 (1)	2.6656 (14)	171 (2)
O4*W*—H42···O2^ii^	0.83 (1)	1.91 (1)	2.7420 (14)	178 (2)
O4*W*—H41···O1^v^	0.83 (1)	1.99 (1)	2.8029 (14)	165 (2)

Symmetry codes: (i) −*x*+1, −*y*+2, −*z*; (ii) −*x*+1, −*y*+2, −*z*+1; (iii) −*x*+1, −*y*+1, −*z*+1; (iv) −*x*+2, −*y*+1, −*z*+1; (v) −*x*+2, −*y*+2, −*z*+1.

### (II) Crystal data

**Table:**

[Co(H_2_O)_6_](C_7_H_5_O_5_S)_2_·2H_2_O	*Z* = 1
*M**_r_* = 605.40	*F*(000) = 313
Triclinic, *P*1	*D*_x_ = 1.746 Mg m^−^^3^
*a* = 6.7774 (11) Å	Mo *K*α radiation, λ = 0.71073 Å
*b* = 6.9866 (11) Å	Cell parameters from 4422 reflections
*c* = 13.721 (2) Å	θ = 3.0–28.3°
α = 91.107 (2)°	µ = 1.01 mm^−^^1^
β = 90.401 (2)°	*T* = 150 K
γ = 117.5832 (19)°	Plate, red-pink
*V* = 575.66 (16) Å^3^	0.30 × 0.14 × 0.08 mm

### (II) Data collection

**Table:**

Bruker Duo with APEXII CCD diffractometer	2578 reflections with *I* > 2σ(*I*)
Radiation source: fine focus sealed tube	*R*_int_ = 0.020
ω scans	θ_max_ = 28.4°, θ_min_ = 3.0°
Absorption correction: multi-scan (SADABS; Krause et al., 2015)	*h* = −9→9
*T*_min_ = 0.688, *T*_max_ = 0.746	*k* = −9→9
8197 measured reflections	*l* = −18→18
2881 independent reflections	

### (II) Refinement

**Table:**

Refinement on *F*^2^	Primary atom site location: dual
Least-squares matrix: full	Secondary atom site location: difference Fourier map
*R*[*F*^2^ > 2σ(*F*^2^)] = 0.024	Hydrogen site location: difference Fourier map
*wR*(*F*^2^) = 0.062	H atoms treated by a mixture of independent and constrained refinement
*S* = 1.05	*w* = 1/[σ^2^(*F*_o_^2^) + (0.030*P*)^2^ + 0.2552*P*] where *P* = (*F*_o_^2^ + 2*F*_c_^2^)/3
2881 reflections	(Δ/σ)_max_ < 0.001
196 parameters	Δρ_max_ = 0.41 e Å^−^^3^
9 restraints	Δρ_min_ = −0.43 e Å^−^^3^

### (II) Special details

**Table:**

Geometry. All esds (except the esd in the dihedral angle between two l.s. planes) are estimated using the full covariance matrix. The cell esds are taken into account individually in the estimation of esds in distances, angles and torsion angles; correlations between esds in cell parameters are only used when they are defined by crystal symmetry. An approximate (isotropic) treatment of cell esds is used for estimating esds involving l.s. planes.

### (II) Fractional atomic coordinates and isotropic or equivalent isotropic displacement parameters (Å^2^)

**Table:**

	*x*	*y*	*z*	*U*_iso_*/*U*_eq_	
Co1	0.500000	0.500000	0.000000	0.01139 (8)	
O1W	0.59207 (18)	0.45162 (17)	0.14109 (8)	0.0168 (2)	
H12	0.490 (3)	0.376 (3)	0.1797 (14)	0.049 (7)*	
H11	0.691 (3)	0.558 (2)	0.1710 (14)	0.033 (6)*	
O3W	0.3051 (2)	0.16486 (17)	−0.01333 (8)	0.0224 (2)	
H32	0.273 (4)	0.088 (3)	−0.0641 (12)	0.051 (7)*	
H31	0.290 (4)	0.086 (3)	0.0345 (11)	0.035 (6)*	
O2W	0.22949 (19)	0.52152 (18)	0.05338 (8)	0.0205 (2)	
H22	0.113 (3)	0.421 (3)	0.0736 (17)	0.051 (7)*	
H21	0.239 (4)	0.632 (2)	0.0817 (15)	0.040 (6)*	
S1	0.15442 (6)	0.93233 (5)	0.23000 (2)	0.01271 (8)	
O1	−0.08728 (17)	0.81710 (17)	0.23196 (8)	0.0193 (2)	
O3	0.25157 (19)	0.89453 (17)	0.14174 (7)	0.0200 (2)	
O4	−0.04855 (19)	0.77393 (18)	0.59079 (8)	0.0204 (2)	
H4A	−0.102 (4)	0.773 (4)	0.6461 (10)	0.049 (7)*	
O5	0.19717 (19)	0.69905 (18)	0.67033 (8)	0.0230 (2)	
O2	0.23686 (17)	1.16534 (16)	0.24926 (7)	0.0157 (2)	
C2	0.1502 (2)	0.8151 (2)	0.41772 (10)	0.0135 (3)	
H2	0.026154	0.842780	0.423772	0.016*	
C5	0.5099 (2)	0.7283 (2)	0.39875 (11)	0.0183 (3)	
H5	0.631065	0.696301	0.392134	0.022*	
C6	0.4323 (2)	0.7927 (2)	0.31792 (11)	0.0165 (3)	
H6	0.500627	0.806287	0.256469	0.020*	
C3	0.2302 (2)	0.7524 (2)	0.49829 (10)	0.0138 (3)	
C7	0.1271 (2)	0.7374 (2)	0.59558 (10)	0.0156 (3)	
C1	0.2530 (2)	0.8369 (2)	0.32839 (10)	0.0132 (3)	
C4	0.4119 (2)	0.7104 (2)	0.48891 (11)	0.0171 (3)	
H4	0.468088	0.669793	0.544051	0.020*	
O4W	−0.15844 (19)	0.20955 (18)	0.12941 (8)	0.0191 (2)	
H42	−0.242 (3)	0.263 (3)	0.1173 (16)	0.037 (6)*	
H41	−0.145 (4)	0.214 (4)	0.1900 (7)	0.045 (7)*	

### (II) Atomic displacement parameters (Å^2^)

**Table:**

	*U*^11^	*U*^22^	*U*^33^	*U*^12^	*U*^13^	*U*^23^
Co1	0.01209 (13)	0.01120 (13)	0.01100 (13)	0.00549 (10)	0.00119 (9)	0.00036 (9)
O1W	0.0156 (5)	0.0169 (5)	0.0132 (5)	0.0035 (4)	0.0007 (4)	0.0009 (4)
O3W	0.0337 (6)	0.0126 (5)	0.0138 (5)	0.0046 (5)	0.0004 (5)	0.0003 (4)
O2W	0.0180 (5)	0.0178 (5)	0.0261 (6)	0.0086 (5)	0.0071 (4)	−0.0011 (5)
S1	0.01491 (17)	0.01270 (16)	0.01041 (16)	0.00627 (13)	0.00180 (12)	0.00044 (12)
O1	0.0148 (5)	0.0194 (5)	0.0195 (5)	0.0046 (4)	−0.0025 (4)	0.0004 (4)
O3	0.0318 (6)	0.0214 (5)	0.0121 (5)	0.0167 (5)	0.0064 (4)	0.0020 (4)
O4	0.0242 (6)	0.0270 (6)	0.0142 (5)	0.0153 (5)	0.0057 (4)	0.0015 (4)
O5	0.0289 (6)	0.0270 (6)	0.0138 (5)	0.0135 (5)	0.0004 (4)	0.0033 (4)
O2	0.0182 (5)	0.0132 (5)	0.0157 (5)	0.0072 (4)	0.0040 (4)	0.0011 (4)
C2	0.0129 (6)	0.0123 (6)	0.0144 (7)	0.0052 (5)	0.0017 (5)	−0.0002 (5)
C5	0.0150 (7)	0.0176 (7)	0.0244 (8)	0.0093 (6)	0.0029 (6)	0.0021 (6)
C6	0.0160 (7)	0.0151 (7)	0.0177 (7)	0.0067 (6)	0.0044 (5)	0.0015 (5)
C3	0.0162 (7)	0.0107 (6)	0.0128 (6)	0.0048 (5)	0.0018 (5)	0.0004 (5)
C7	0.0184 (7)	0.0102 (6)	0.0156 (7)	0.0046 (5)	0.0010 (5)	0.0000 (5)
C1	0.0140 (6)	0.0113 (6)	0.0129 (6)	0.0047 (5)	0.0010 (5)	0.0002 (5)
C4	0.0178 (7)	0.0140 (7)	0.0194 (7)	0.0073 (6)	−0.0014 (5)	0.0022 (5)
O4W	0.0223 (6)	0.0231 (5)	0.0152 (5)	0.0134 (5)	−0.0012 (4)	−0.0005 (4)

### (II) Geometric parameters (Å, º)

**Table:**

Co1—O2W	2.0470 (11)	O4—C7	1.3298 (18)
Co1—O2W^i^	2.0470 (11)	O4—H4A	0.841 (10)
Co1—O3W	2.0921 (11)	O5—C7	1.2126 (18)
Co1—O3W^i^	2.0921 (11)	C2—C1	1.3898 (19)
Co1—O1W	2.1107 (11)	C2—C3	1.3921 (19)
Co1—O1W^i^	2.1107 (11)	C2—H2	0.9500
O1W—H12	0.844 (10)	C5—C4	1.389 (2)
O1W—H11	0.835 (9)	C5—C6	1.393 (2)
O3W—H32	0.836 (10)	C5—H5	0.9500
O3W—H31	0.839 (9)	C6—C1	1.393 (2)
O2W—H22	0.832 (10)	C6—H6	0.9500
O2W—H21	0.830 (10)	C3—C4	1.398 (2)
S1—O1	1.4534 (11)	C3—C7	1.494 (2)
S1—O3	1.4594 (11)	C4—H4	0.9500
S1—O2	1.4735 (10)	O4W—H42	0.827 (10)
S1—C1	1.7742 (14)	O4W—H41	0.834 (10)
			
O2W—Co1—O2W^i^	180.0	O1—S1—C1	106.70 (7)
O2W—Co1—O3W	88.95 (5)	O3—S1—C1	106.69 (7)
O2W^i^—Co1—O3W	91.05 (5)	O2—S1—C1	106.26 (6)
O2W—Co1—O3W^i^	91.05 (5)	C7—O4—H4A	112.1 (17)
O2W^i^—Co1—O3W^i^	88.95 (5)	C1—C2—C3	119.60 (13)
O3W—Co1—O3W^i^	180.0	C1—C2—H2	120.2
O2W—Co1—O1W	91.15 (4)	C3—C2—H2	120.2
O2W^i^—Co1—O1W	88.85 (4)	C4—C5—C6	120.60 (13)
O3W—Co1—O1W	87.56 (4)	C4—C5—H5	119.7
O3W^i^—Co1—O1W	92.44 (4)	C6—C5—H5	119.7
O2W—Co1—O1W^i^	88.85 (4)	C5—C6—C1	119.12 (13)
O2W^i^—Co1—O1W^i^	91.15 (4)	C5—C6—H6	120.4
O3W—Co1—O1W^i^	92.44 (4)	C1—C6—H6	120.4
O3W^i^—Co1—O1W^i^	87.56 (4)	C2—C3—C4	120.01 (13)
O1W—Co1—O1W^i^	180.0	C2—C3—C7	120.06 (13)
Co1—O1W—H12	118.4 (17)	C4—C3—C7	119.90 (13)
Co1—O1W—H11	117.6 (15)	O5—C7—O4	123.99 (14)
H12—O1W—H11	110 (2)	O5—C7—C3	123.95 (14)
Co1—O3W—H32	127.8 (18)	O4—C7—C3	112.06 (12)
Co1—O3W—H31	120.3 (15)	C2—C1—C6	120.86 (13)
H32—O3W—H31	108 (2)	C2—C1—S1	117.65 (11)
Co1—O2W—H22	127.0 (17)	C6—C1—S1	121.45 (11)
Co1—O2W—H21	122.7 (16)	C5—C4—C3	119.79 (13)
H22—O2W—H21	105 (2)	C5—C4—H4	120.1
O1—S1—O3	114.70 (7)	C3—C4—H4	120.1
O1—S1—O2	111.06 (6)	H42—O4W—H41	106 (2)
O3—S1—O2	110.90 (6)		
			
C4—C5—C6—C1	−0.7 (2)	C5—C6—C1—S1	176.87 (11)
C1—C2—C3—C4	−0.4 (2)	O1—S1—C1—C2	−44.42 (13)
C1—C2—C3—C7	177.42 (12)	O3—S1—C1—C2	−167.47 (11)
C2—C3—C7—O5	−174.55 (14)	O2—S1—C1—C2	74.15 (12)
C4—C3—C7—O5	3.3 (2)	O1—S1—C1—C6	137.89 (12)
C2—C3—C7—O4	4.61 (19)	O3—S1—C1—C6	14.84 (14)
C4—C3—C7—O4	−177.56 (13)	O2—S1—C1—C6	−103.54 (12)
C3—C2—C1—C6	1.3 (2)	C6—C5—C4—C3	1.5 (2)
C3—C2—C1—S1	−176.42 (10)	C2—C3—C4—C5	−1.0 (2)
C5—C6—C1—C2	−0.7 (2)	C7—C3—C4—C5	−178.82 (13)

Symmetry code: (i) −*x*+1, −*y*+1, −*z*.

### (II) Hydrogen-bond geometry (Å, º)

**Table:**

*D*—H···*A*	*D*—H	H···*A*	*D*···*A*	*D*—H···*A*
O1*W*—H12···O2^ii^	0.84 (1)	1.94 (1)	2.7757 (15)	170 (2)
O1*W*—H11···O1^iii^	0.84 (1)	1.91 (1)	2.7382 (15)	175 (2)
O3*W*—H32···O4*W*^iv^	0.84 (1)	2.04 (2)	2.7887 (16)	150 (2)
O3*W*—H31···O3^ii^	0.84 (1)	1.95 (1)	2.7852 (16)	178 (2)
O2*W*—H22···O4*W*	0.83 (1)	1.92 (1)	2.7516 (16)	174 (2)
O2*W*—H21···O3	0.83 (1)	1.96 (1)	2.7925 (15)	176 (2)
O4—H4*A*···O2^v^	0.84 (1)	1.86 (1)	2.6703 (15)	162 (2)
O4*W*—H42···O1*W*^vi^	0.83 (1)	2.12 (1)	2.8960 (16)	158 (2)
O4*W*—H41···O5^vii^	0.83 (1)	2.08 (1)	2.8503 (16)	153 (2)

Symmetry codes: (ii) *x*, *y*−1, *z*; (iii) *x*+1, *y*, *z*; (iv) −*x*, −*y*, −*z*; (v) −*x*, −*y*+2, −*z*+1; (vi) *x*−1, *y*, *z*; (vii) −*x*, −*y*+1, −*z*+1.

## References

[bb1] Abdelhak, J., Namouchi Cherni, S. & Jouini, T. (2005). *Z. Kristallogr.* **220**, 183–184.

[bb2] Bettinger, R. T., Squattrito, P. J. & Aulakh, D. (2020). *Acta Cryst.* E**76**, 1275–1278. 10.1107/S2056989020009408 PMC7405583 32844013

[bb3] Bruker (2015). *APEX3* and *SAINT*. Bruker AXS Inc., Madison, Wisconsin, USA.

[bb4] Cai, J. (2004). *Coord. Chem. Rev.* **248**, 1061–1083.

[bb5] Cotton, F. A., Daniels, L. M., Murillo, C. A. & Quesada, J. F. (1993). *Inorg. Chem.* **32**, 4861–4867.

[bb6] Dey, C., Kundu, T., Biswal, B. P., Mallick, A. & Banerjee, R. (2014). *Acta Cryst.* B**70**, 3–10. 10.1107/S2052520613029557 24441122

[bb7] Gao, S., Zhu, Z.-B., Huo, L.-H. & Ng, S. W. (2005*a*). *Acta Cryst.* E**61**, m279–m281.

[bb8] Gao, S., Zhu, Z.-B., Huo, L.-H. & Ng, S. W. (2005*b*). *Acta Cryst.* E**61**, m517–m518.

[bb9] Groom, C. R., Bruno, I. J., Lightfoot, M. P. & Ward, S. C. (2016). *Acta Cryst.* B**72**, 171–179. 10.1107/S2052520616003954 PMC4822653 27048719

[bb10] Krause, L., Herbst-Irmer, R., Sheldrick, G. M. & Stalke, D. (2015). *J. Appl. Cryst.* **48**, 3–10. 10.1107/S1600576714022985 PMC4453166 26089746

[bb11] Lamshöft, M., Storp, J., Ivanova, B. & Spiteller, M. (2011). *Polyhedron*, **30**, 2564–2573.

[bb12] Leonard, M. A., Squattrito, P. J. & Dubey, S. N. (1999). *Acta Cryst.* C**55**, 35–39.

[bb13] Li, L.-Z., Xu, T., Wang, D.-Q. & Niu, M.-J. (2004). *Acta Cryst.* E**60**, m1374–m1375.

[bb14] Ma, J.-F., Yang, J. & Liu, J.-F. (2003*a*). *Acta Cryst.* E**59**, m483–m484.

[bb15] Ma, J.-F., Yang, J. & Liu, J.-F. (2003*b*). *Acta Cryst.* E**59**, m481–m482. 10.1107/s0108270103022443 14605398

[bb16] Ma, J.-F., Yang, J. & Liu, J.-F. (2003*c*). *Acta Cryst.* E**59**, m487–m488.

[bb17] Ma, J.-F., Yang, J. & Liu, J.-F. (2003*d*). *Acta Cryst.* E**59**, m478–m480.

[bb18] Ma, J.-F., Yang, J. & Liu, J.-F. (2003*e*). *Acta Cryst.* E**59**, m485–m486.

[bb19] Palmer, D. (2014). *CrystalMaker*. CrystalMaker Software Ltd, Yarnton, England.

[bb20] Prochniak, G., Videnova-Adrabinska, V., Daszkiewicz, M. & Pietraszko, A. (2008). *J. Mol. Struct.* **891**, 178–183. 10.1107/S0108270107025152 17609580

[bb21] Senevirathna, D. C., Werrett, M. V., Blair, V. L., Mehring, M. & Andrews, P. C. (2018). *Chem. Eur. J.* **24**, 6722–6726. 10.1002/chem.201705981 29532528

[bb22] Sheldrick, G. M. (2015*a*). *Acta Cryst.* A**71**, 3–8.

[bb23] Sheldrick, G. M. (2015*b*). *Acta Cryst.* C**71**, 3–8.

[bb24] Shimizu, G. K. H., Vaidhyanathan, R. & Taylor, J. M. (2009). *Chem. Soc. Rev.* **38**, 1430–1449. 10.1039/b802423p 19384446

[bb25] Song, J. H., Kim, D. W., Kang, D. W., Lee, W. R. & Hong, C. S. (2019). *Chem. Commun.* **55**, 9713–9716. 10.1039/c9cc04474d 31353388

[bb26] Squattrito, P. J., Lambright-Mutthamsetty, K. J., Giolando, P. A. & Kirschbaum, K. (2019). *Acta Cryst.* E**75**, 1801–1807. 10.1107/S2056989019014610 PMC6829707 31709112

